# Editorial: Exploring the impact of biologics in nephrology: clinical advances and future perspectives

**DOI:** 10.3389/fmed.2025.1697374

**Published:** 2025-09-29

**Authors:** Marta Calatroni, Manuel A. Podestà, David Cucchiari, Francesco Reggiani

**Affiliations:** ^1^Department of Biomedical Sciences, Humanitas University, Milan, Italy; ^2^Nephrology and Dialysis Division, IRCCS Humanitas Research Hospital, Milan, Italy; ^3^Unit of Nephrology, Dialysis and Renal Transplantation, Fondazione IRCCS Ca' Granda Ospedale Maggiore Policlinico, Milan, Italy; ^4^Department of Nephrology and Kidney, Transplantation Hospital Clinic, Barcelona, Spain

**Keywords:** biologics, glomerular diseases, minimal change disease, rituximab, B-cell depletion, Infection risk

## Introduction

The advent of biologic therapies has reshaped the management of immune-mediated kidney diseases. Among them, rituximab, a chimeric monoclonal antibody targeting CD20, has become the cornerstone of treatment in several nephrological conditions. Two recent studies published in *Frontiers in Immunology* provide insights into rituximab use in glomerulonephritis. Wang et al. investigated rituximab as initial therapy for autoimmune podocytopathies with a minimal change disease (MCD) histologic pattern, confirming previous evidence on B cell-depletion as a first-line alternative to glucocorticoids. Conversely, Xu et al. examined severe infections risk following rituximab in patients with primary vs. secondary glomerulopathies. Together, these studies illustrate both promise and challenges of rituximab in nephrology.

## Rituximab as first-line therapy in autoimmune podocytopathies

Primary podocytopathies with minimal-change lesions account for 10%−15% of adult nephrotic syndrome and remains the leading cause of nephrotic syndrome in children. Glucocorticoids induce remission in most pediatric cases, whereas in adults responses are less consistent and require prolonged courses. Despite efficacy, treatment is limited by substantial toxicity, highlighting the need for alternatives (1).

Recent studies in primary podocytopathies have identified antibodies against slit diaphragm proteins (e.g., nephrin, podocin, KIRREL1), closely linked to disease activity, reinforcing the concept of primary podocytopathies as B-cell–mediated autoimmune disorders and supporting B-cell–targeted strategies (2).

Rituximab has long been used in autoimmune podocytopathies as a steroid-sparing agent rather than as first-line therapy (1). However, its modulation on anti-slit antibody responses has sparked interest in its upfront use. Case series reported complete remission rates from 55 to 89% with rituximab monotherapy (3). By contrast, a retrospective study directly comparing rituximab with glucocorticoids found complete remission in only 50% of rituximab-treated patients, compared with 96% with steroids (4). This raises concerns about its efficacy as monotherapy in the first-line setting.

In their study, Wang et al. compared rituximab with glucocorticoids in 28 patients with biopsy-proven MCD (14 receiving rituximab). Propensity score matching ensured comparable baseline characteristics. Average age was 35.93 ± 16.66 years. All patients achieved remission at 24 weeks, with no significant difference in remission, time to remission, or relapse rates. Both groups showed improved proteinuria, albumin, and stable renal function. Rituximab was well tolerated, with only one non-serious infusion reaction, while the glucocorticoid group exhibited typical steroid side effects. These findings, although from a small cohort, question the inevitability of steroids as first-line therapy. Among limitations, the absence of testing for anti-slit diaphragm antibodies precluded a definitive diagnosis of autoimmune podocytopathy; however, the observed response to rituximab supports a B-cell–mediated process. That rituximab can induce remission with comparable efficacy but fewer metabolic side effects justifies randomized controlled trials as alternative induction, particularly in patients at high risk of steroid toxicity.

## Infection risk after rituximab: primary vs. secondary nephropathy

If efficacy in autoimmune podocytopathies suggests a role for rituximab earlier in treatment algorithms, safety—particularly infection risk—remains a critical consideration in the use of B-cell–depleting therapies. The retrospective study by Xu et al. distinguished infection risks between primary and secondary nephropathies.

Among 123 rituximab-treated patients followed for nearly 20 months, 26% developed severe infections. Patients with secondary nephropathies (predominantly lupus nephritis and ANCA vasculitis) had higher risk than primary diseases (membranous nephropathy, podocytopathies). The hazard ratio for serious infection in secondary vs. primary nephropathy was 5.86 (95% CI: 1.05–32.63). Risk factors included advanced age, prior infection, and low IgG levels. Respiratory infections predominated, with bacteria as the most common pathogens, but opportunistic infections, including *Pneumocystis jirovecii* pneumonia, pulmonary aspergillosis, herpes zoster, and tuberculosis, were also observed. This study highlights the need for risk stratification. While primary nephropathies patients appear relatively protected, systemic autoimmune conditions and renal involvement carry a higher infectious burden. Practical implications include routine IgG assessment, evaluation of infection history, and prophylaxis (TMP-SMX, antiviral or antifungal coverage in selected cases) as part of rituximab protocols.

## Reconciling efficacy and safety and future perspectives

Together, these two studies illustrate the dual narrative of biologics in nephrology (Wang et al., Xu et al.). On one hand, rituximab may redefine therapeutic algorithms in diseases traditionally treated with steroids, as exemplified by autoimmune podocytopathies. On the other hand, benefits must be balanced against safety, particularly in systemic autoimmune diseases where infection risk is substantial. This juxtaposition raises key questions for future practice. Should rituximab be formally tested against glucocorticoids in large randomized controlled trials (RCT) as initial therapy for autoimmune podocytopathies and which patient subgroups would stand to have more benefit? No RCT have yet addressed this question and closing this evidence gap is essential to determine whether rituximab can truly replace glucocorticoids as a first-line regimen. Can infection risk be more precisely stratified with baseline IgG levels, comorbidities, and prior infection history, to enable tailored prophylactic strategies? Moreover, can dosing regimens be optimized to preserve efficacy while limiting immunosuppression? Finally, might newer anti-CD20 antibodies or alternative B-cell–targeting agents improve outcomes while reducing infectious?

## Conclusion

The trajectory of biologics in nephrology mirrors that of oncology and rheumatology: initial adoption in refractory cases, followed by gradual movement toward earlier lines of therapy. Rituximab may represent only the first wave. Humanized or fully human anti-CD20 antibodies (obinutuzumab, ofatumumab), complement inhibitors, and plasma cell-targeting agents are entering clinical evaluation, each raising new questions about optimal integration and long-term safety.

In this context, the lessons from Wang et al. and Xu et al. are useful. Rituximab exemplifies both the promise and the perils of this shift. For autoimmune podocytopathies, rituximab may allow steroid-sparing management, particularly relevant for patients at high risk of steroid systemic toxicity. Especially for secondary nephropathies, vigilance is warranted, and precision approaches integrating biomarkers of immune competence may help identify patients at unacceptable infection risk. As the field advances, nephrology must embrace a precision medicine framework, matching biologics not only to disease mechanisms but also to individual patient risk profiles. Only through this dual focus on efficacy and safety can biologics truly transform outcomes for patients with kidney disease.
